# Annotations

**Published:** 1902-03-22

**Authors:** 


### ANNOTATIONS.

### Small-pox and Vaccination.

Few things could be more curious than the disproportion between what may be described as the "small-pox scare" now prevalent and the effectiveness of the preventive measures which are being enforced by public authorities. While private individuals have been suffering under alarm for their own safety, while the heads of great business houses in London have been offering the alternatives of revaccination or dismissal to all the persons in their employment, and while newspapers have been wondering how far the fact that, in round numbers, one out of every 3,000 of the population of the County of London is at this moment ill with small-pox, will be likely to interfere with the number of visitors for the Coronation, we learn from a memorandum just issued by Dr. Seaton, the vigilant M. O. H. for Surrey, that the Borough of Croydon and parts of the adjacent Rural district were rather sharply attacked during the month of February, the number of cases having been 17, as compared with 5 in the two previous months. Some of the most troublesome outbreaks, Dr. Seaton continues, have been due to direct importation from Dartford, and have resulted from the non-vaccination of persons who have been exposed to every possible chance of infection. It is, he justly adds, surprising and alarming to hear of numbers of persons being employed in connection with the extension of the small-pox hospitals, without this protection being in some way secured. In the meanwhile, several beds at the Cheam Hospital are occupied by cases of the kind, that is to say by workmen who have been employed at the Dartford Hospitals without being first revaccinated, who have contracted the disease there, and have been sent back to their homes during its early stage. But the mischief may spread much further. We have been informed quite lately that a workman engaged on the hospital extension at Dartford, feeling ill, went home to his mother at Cambridge, where his illness turned out to be small-pox. And his mother was a bed-maker at one of the largest colleges! We do not know, of course, who is responsible for the employment in or about the Dartford hospitals of the unprotected workmen referred to, or for permitting them to return to various parts of the suburbs as soon as the natural consequences of their exposure to infection are produced; but there can be no doubt that the responsibility, upon whatever shoulders it ought to fall, can hardly be regarded as other than a criminal one. The precautions which are enforced, for their own sakes, with regard to nurses at small-pox hospitals, should certainly be enforced still more strictly, for the sake also of the public safety, in the cases of workmen who are employed about the buildings, and who will be certain, if themselves attacked, to return straightway to their homes.

### A Labour Colony.

A few years ago there was established at Ruthwell, in Dumfriesshire, a labour colony, the intention of which was to give temporary shelter to the genuine unemployed. During last year a hundred men passed through it, men drawn from different trades, and possessing different capacities, and the result seems to have been satisfactory. Among the inmates were schoolmasters, commercial travellers, clerks, tailors, masons, carpenters, plumbers, slaters, painters, and common labourers. It is likely that some of the first-mentioned classes, whatever were the faults or misfortunes which made them seek the shelter of the colony, had a little to endure from the habits of some of their neighbours, but the superintendent reports that, considering the variety of characters and temperaments, the conduct of the colonists has been very good. Occasionally it has been necessary to dismiss an inmate for idleness or bad conduct, but on the whole they have shown themselves willing to work and amenable to discipline. The average age of the men admitted was 35, for the colony is meant only to be a temporary shelter for men who, after a comparatively short stay there, will again be able to earn their living. Of those who left the colony during the year the majority secured situations, while 36 left to push their way in the world again, and seven emigrated to Canada. The superintendent, who keeps up his interest in those who have once been under his charge, says that from the letters he has received many of those who went to situations from the colony are prospering. With men of this stamp emigration seems a very safe experiment.

### Public Libraries and Infection.

The Lambeth Board of Guardians have decided to establish a free library for the nurses in the workhouse infirmary, in order to take away the temptation to bring in books from outside libraries during the present epidemic of small-pox. This decision would have pleased Mr. Ruskin, who denounced the idea of borrowing books from public libraries as "foolish and filthy." It must be owned that the library book is a possible source of danger. It is rather strange how people who have the greatest terror of infection will take books from a library without thought of risk, and equally strange and far more reprehensible that some will borrow a book from a library to while away the hours when nursing a fever or small-pox patient, or even to amuse the patient during the long hours of convalescence. Indeed, considering the carelessness of the public in this matter alone, the wonder is, not that a book occasionally carries infection, but that it so rarely does so. Of course the Lambeth Guardians will have the ratepayers to reckon with, and these may think that they are in no way bound to pay for the reading with which the nurses amuse their leisure hours; but that is a matter for their own consideration. It must be said, however, that now that cheap editions of good books are so many, there is not the same need for the provision of light literature in public libraries, or such semi-public ones as that in the Lambeth Workhouse Infirmary. At an expenditure of sixpence each, the nurses might provide themselves with a library of good and interesting fiction, and probably all of them pay a great deal more in the course of a year, or even a quarter, in library fees. They could exchange the books with each other, and thus the sixpences of each would cover a good deal of ground.

